# Comparison of two bundles for reducing surgical site infection in colorectal surgery: multicentre cohort study

**DOI:** 10.1093/bjsopen/zrae080

**Published:** 2024-08-06

**Authors:** Miriam Flores-Yelamos, Aina Gomila-Grange, Josep M Badia, Alexander Almendral, Ana Vázquez, David Parés, Marta Pascual, Enric Limón, Miquel Pujol, Montserrat Juvany, members of the VINCat Colorectal Surveillance Team, Domenico Fraccalvieri, Ana Abad-Torrent, Alejandro Solís-Peña, Mireia Puig-Asensio, Lucrecia López, Marta Piriz, Mercè Hernández, Dolors Castellana, Elisa Montiu González, Graciano García Pardo, Francesc Feliu Villaró, Josep Rebull Fatsini, Marie France Domènech Spaneda, Marta Conde Galí, Anna Oller Pérez-Hita, Lydia Martín, Ana Lerida, Sebastiano Biondo, Emilio Jiménez Martínez, Nieves Sopena Galindo, Ignasi Camps Ausàs, Carmen Ferrer, Luis Salas, Rafael Pérez Vidal, Dolors Mas Rubio, Irene García de la Red, Mª Angels Iruela Castillo, Eva Palau i Gil, José Antonio Martínez, M Blanca Torralbo Navarro, Maria López, Carol Porta, Alex Smithson Amat, Guillen Vidal Escudero, José Carlos de la Fuente Redondo, Montse Rovira Espés, Arantxa Mera Fidalgo, Luis Escudero Almazán, Monserrat Ortega Raya, Mª Carmen Álvarez Moya, Vicens Diaz-Brito, Laura Grau Palafox, Yésika Angulo Gómez, Anna Besolí Codina, Carme Autet Ricard, Carlota Hidalgo López, Elisabeth Lerma-Chippirraz, Demelza Maldonado López, David Blancas, Esther Moreno Rubio, Roser Ferrer i Aguilera, Simona Iftimie, Antoni Castro-Salomó, Rosa Laplace Enguídanos, Maria Carmen Sabidó Serra, Núria Bosch Ros, Virginia Pomar Solchaga, Laura Lázaro Garcia, Angeles Boleko Ribas, Jordi Palacín Luque, Alexandra Lucía Moise, Mª Carmen Fernández Palomares, Santiago Barba Sopeña, Eduardo Sáez Huertas, Sara Burges Estada, Josep María Tricas Leris, Eva Redon Ruiz, Montse Brugués, Susana Otero Aced, Maria Cuscó Esteve, Francisco José Vargas-Machuca, Mª de Gracia García Ramírez, Ana Maria Ciscar Bellés, Elena Vidal Díez, Mariló Marimón Morón, Marisol Martínez Sáez, Josep Farguell, Mireia Saballs, Montserrat Vaqué Franco, Leonor Invernón Garcia, Rosa Laplace Enguídanos, Meritxell Guillemat Marrugat, Ana Coloma Conde

**Affiliations:** Department of Surgery, Hospital General de Granollers, Granollers, Spain; School of Medicine, Universitat Internacional de Catalunya, Sant Cugat del Vallès, Barcelona, Spain; Department of Infectious Diseases, Hospital Universitari Parc Taulí, Sabadell, Spain; Department of Surgery, Hospital General de Granollers, Granollers, Spain; School of Medicine, Universitat Internacional de Catalunya, Sant Cugat del Vallès, Barcelona, Spain; Surveillance of Healthcare Related Infections in Catalonia Programme, VINCat, Departament de Salut, Generalitat de Catalunya, Catalonia, Spain; Servei d'Estadística Aplicada, Universitat Autònoma de Barcelona, Bellaterra, Barcelona, Spain; Colorectal Surgery Unit, Department of Surgery, Hospital Universitari Germans Trias i Pujol, Universitat Autónoma de Barcelona, Badalona, Barcelona, Spain; Department of Surgery, Hospital del Mar, Barcelona, Spain; Surveillance of Healthcare Related Infections in Catalonia Programme, VINCat, Departament de Salut, Generalitat de Catalunya, Catalonia, Spain; Department of Public Health, Mental Health & Mother–Infant Nursing, Faculty of Nursing, University of Barcelona, Barcelona, Spain; Centro de Investigación Biomédica en Red de Enfermedades Infecciosas (CIBERINFEC), Instituto de Salud Carlos III, Madrid, Spain; Surveillance of Healthcare Related Infections in Catalonia Programme, VINCat, Departament de Salut, Generalitat de Catalunya, Catalonia, Spain; Centro de Investigación Biomédica en Red de Enfermedades Infecciosas (CIBERINFEC), Instituto de Salud Carlos III, Madrid, Spain; Department of Infectious Diseases, Hospital Universitari de Bellvitge—IDIBELL, L’Hospitalet de Llobregat, Spain; Department of Surgery, Hospital del Mar, Barcelona, Spain

## Abstract

**Background:**

There is controversy regarding the maximum number of elements that can be included in a surgical site infection prevention bundle. In addition, it is unclear whether a bundle of this type can be implemented at a multicentre level.

**Methods:**

A pragmatic, multicentre cohort study was designed to analyse surgical site infection rates in elective colorectal surgery after the sequential implementation of two preventive bundle protocols. Secondary outcomes were to determine compliance with individual measures and to establish their effectiveness, duration of stay, microbiology and 30-day mortality rate.

**Results:**

A total of 32 205 patients were included. A 50% reduction in surgical site infection was achieved after the implementation of two sequential sets of bundles: from 18.16% in the Baseline group to 10.03% with Bundle-1 and 8.19% with Bundle-2. Bundle-2 reduced superficial-surgical site infection (OR 0.74 (95% c.i. 0.58 to 0.95); *P* = 0.018) and deep-surgical site infection (OR 0.66 (95% c.i. 0.46 to 0.93); *P* = 0.018) but not organ/space-surgical site infection (OR 0.88 (95% c.i. 0.74 to 1.06); *P* = 0.172). Compliance increased after the addition of four measures to Bundle-2. In the multivariable analysis, for organ/space-surgical site infection, laparoscopy, oral antibiotic prophylaxis and mechanical bowel preparation were protective factors in colonic procedures, while no protective factors were found in rectal surgery. Duration of stay fell significantly over time, from 7 in the Baseline group to 6 and 5 days for Bundle-1 and Bundle-2 respectively (*P* < 0.001). The mortality rate fell from 1.4% in the Baseline group to 0.59% and 0.6% for Bundle-1 and Bundle-2 respectively (*P* < 0.001). There was an increase in Gram-positive bacteria and yeast isolation, and reduction in Gram-negative bacteria and anaerobes in organ/space-surgical site infection.

**Conclusions:**

The addition of measures to create a final 10-measure protocol had a cumulative protective effect on reducing surgical site infection. However, organ/space-surgical site infection did not benefit from the addition. No protective measures were found for organ/space-surgical site infection in rectal surgery. Compliance with preventive measures increased from Bundle-1 to Bundle-2.

## Teaser

A pragmatic, multicentre cohort study was designed to analyse surgical site infection rates in elective colorectal surgery after sequential application of two preventive bundle protocols. A 50% reduction in surgical site infections was achieved in a cohort of 32 205 patients after sequential application of the bundles. The addition of four supplementary measures to create a final 10-measure protocol increased compliance, but only incisional surgical site infections benefited from the addition, while organ/space-surgical site infections did not.

## Introduction

Surgical site infection (SSI) is one of the most common healthcare-related infections in Europe and also the most prevalent postsurgical complication^1,2^. Although its incidence has fallen, it remains a significant healthcare concern due to its impact on hospital stay, antibiotic consumption, readmission and reoperation rates. It also impacts patients’ outcomes by increasing the morbidity rate and reducing survival^1,3–5^.

Colorectal surgery has the highest SSI rates of all surgical interventions, with a reported incidence of up to 26% compared with overall surgical rates below 6%^2^. It has been estimated that about 60% of SSIs are preventable^6,7^. However, implementation of different preventative strategies has shown varying rates of success. In this context, the implementation of epidemiological surveillance programmes and preventive bundles has emerged as a promising strategy.

Bundles comprise limited sets of easy-to-implement and evidence-based preventive measures which, applied together, improve patients’ outcomes. Designing and implementing bundles can be challenging; some have proven effectiveness in colorectal surgery^8,9^, but others do not^10^. Most interventions impact on superficial SSI (S-SSI) rates and have less impact on deep (D-SSIs) and organ/space-SSI (O/S-SSIs)^11–13^. Bundles may be relatively easy to introduce at single centres, but there is less evidence of the effectiveness of their implementation in large groups of hospitals^14,15^. In this context, the successful implementation of bundles with small numbers of measures may take more than 4 years^16^.

It has been argued that increasing the number of interventions in a bundle reduces compliance. However, two meta-analyses on colorectal surgery found that bundles containing 11 elements or more demonstrated the greatest reduction in SSIs^9,17^.

This study aimed to better understand the impact of adding new measures to an established bundle within a nationwide surveillance programme. Two bundles were compared to measure the effectiveness of each specific measure. One bundle comprised six measures and the other comprised 10 and were implemented sequentially in a large series of elective colorectal procedures from 2011 to 2022.

The hypothesis was that thorough introduction of a well designed, large bundle of best practice preventive measures would achieve good adherence and would reduce SSI rates after colorectal surgery.

## Methods

### Setting and patients

This pragmatic, multicentre cohort study comprised a network of 65 public and private hospitals that prospectively record data in order to reduce SSI rates and to improve other healthcare outcomes in elective colorectal surgery. The infection control team (ICT) at each hospital performed prospective surveillance to ensure adequate data collection with a minimum mandatory follow-up of 30 days after surgery, an electronic review of clinical records to record readmissions, visits to the emergency department or other healthcare facilities, and microbiological and radiological data. The data analysis and results were carried out retrospectively.

Patients who underwent elective colorectal surgery between January 2011 and December 2022 were included. Patients with wound class 2 (clean-contaminated) and 3 (contaminated), according to the National Healthcare Safety Network Classification^18^, were monitored. Patients with wound class 4 (peritonitis) and with previous ostomies were excluded. *Table S1* shows the inclusion and exclusion criteria for colorectal surgery surveillance in detail.

Three sequential phases were compared: a baseline interval before bundle implementation (Baseline group), from January 2011 to June 2016; a Bundle-1 interval after the implementation of a six-measure bundle (Bundle-1 group), from July 2016 to June 2018; and a Bundle-2 interval after the implementation of a 10-measure bundle (Bundle-2 group), from July 2018 to December 2022 (*Fig. S1*).

During the baseline interval, before the introduction of each bundle, detailed operational definition documents were generated annually and shared with all hospitals in the network, together with the annual performance benchmark. The implementation phases of each bundle began 3 months before the start, with dissemination of the recommended measures by e-mail to all participating hospitals, posting of the procedure manual on the surveillance system website, and a workshop for infection control groups from all hospitals, including surgeons, anaesthetists, surgical nursing teams and the ICT itself.

The definitions, criteria and surveillance methodology used by the ICT staff were identical in all three study intervals. ICTs were pretrained to ensure consistent and accurate data collection, and audits of the data provided were conducted at different points in the programme's development. A programme of continuing education for ICTs was also maintained throughout the surveillance programme, and personalized counselling was provided to ICTs when the SSI diagnosis was doubtful or other operational problems occurred. Mandatory active surveillance after discharge was conducted until postoperative day 30.

### Data source, definitions, study outcomes and variables

The data were taken from the surveillance programme of healthcare-associated infection in Catalonia, Spain (VINCat), which performs prospective and interventional surveillance of SSIs at public and private hospitals.

The primary outcome was the development of an SSI according to the Centres for Disease Control (CDC) definitions within 30 days after surgery^19^. Incisional (I-SSI) includes S-SSI (skin and subcutaneous tissue involvement) and D-SSI (affects deep soft tissues), while O/S-SSI affects any anatomical structure other than the incision^19^. O/S-SSI is associated with a higher mortality rate and higher healthcare costs^20^.

Secondary outcomes were to determine compliance with individual measures and their effectiveness, assessment of duration of stay (LOS), 30-day mortality rate and SSI-causing microorganisms.

Routine demographic data collected by the surveillance system were analysed, including age, sex, American Society of Anesthesiologists (ASA) surgical risk score, information on the surgical procedure (including open, laparoscopic or robotic approach), wound contamination class and duration of surgery. The term minimally invasive surgery (MIS) includes procedures performed by laparoscopic and robotic surgery. The National Nosocomial Infection Surveillance (NNIS) score was also calculated for each patient.

As a source of data on compliance with the measures included in the bundles, a checklist of prevention measures was generated for each bundle. The data from these checklists were prospectively transmitted online to the centralized database of the surveillance programme. The criteria used to consider antibiotic prophylaxis ‘adequate’ included: the type of drug, the dose administered, the timing of infusion, its completion before the surgical incision and the duration of therapy. A single deviation from the recommended guidelines was enough to consider the process inadequate.

### Intervention

In the Baseline group, certain measures such as intravenous antibiotic prophylaxis and the use of laparoscopy were already included as standard clinical practices. In Bundle-1, six specific colorectal measures were recommended: intravenous antibiotic prophylaxis, laparoscopy, oral antibiotic prophylaxis (OAP), mechanical bowel preparation (MBP), maintenance of normothermia and double-ring plastic wound retractor. In Bundle-2, four additional general measures were incorporated: adequate hair removal, skin antisepsis with 2% chlorhexidine gluconate alcohol solution (CHG-alcohol), perioperative glucose monitoring and changing of instruments before wound closure. The measures implemented are described in *Table S2*.

### Statistical analysis

Data were summarized using frequencies and proportions for categorical variables and means with standard deviation or medians with interquartile range for continuous variables, depending on the distribution. The infection rate was expressed as the crude percentage of operations resulting in SSI per number of surgical procedures. To address confounding variables and to minimize selection bias among the three groups, inverse probability of treatment weighting (IPTW) was used^21,22^. Preweighted groups were compared using Pearson's chi-square test or Fisher's exact test for categorical variables and Student's *t*-test or ANOVA for continuous variables. The effectiveness of IPTW in achieving a balance between confounding variables was assessed by comparing standardized differences between groups before and after weighting^23^. The comparative assessment of outcomes between groups used univariate logistic regression for categorical outcomes and the Wilcoxon rank-sum test for continuous outcomes. Additionally, a univariable and multivariable logistic regression model based on unweighted cluster data was used to characterize the effect of specific measures on SSIs. The results of the logistic regression model were presented in terms of odds ratios (OR) along with their corresponding 95% confidence intervals (95% c.i.). The significance level was set at 0.05 for all tests. All results were analysed using R v4.2.2 software by The R Foundation, Vienna, Austria^24^.

### Ethical issues

The data are stored in a large, non-publicly available national database. The study is reported in accordance with the Strengthening the Reporting of Observational Studies in Epidemiology (STROBE) statement: guidelines for reporting observational studies^25^, and was approved by the Clinical Research Ethics Committee of the Hospital General de Granollers, which considered that informed consent was not necessary given that the data were anonymized, and the confidentiality of all patients was maintained (code 20222022). The project was registered at clinicaltrials.gov as NCT06244836.

### Institutional review board statement

Data extraction was approved by the Institutional Research Board with code 20166009, and the study was approved by the Clinical Research Ethics Committee of Hospital General de Granollers, with code 2021006. The need for informed consent and the provision of an information sheet were waived because data were routinely collected as part of hospital surveillance and quality improvement.

## Results

A total of 32 205 patients were included: 18 664 in the Baseline group, 3908 in the Bundle-1 group and 9633 in the Bundle-2 group. Demographic and surgical characteristics are shown in *Table 1*. After the implementation of IPTW, an assessment of the balance of the variables among the groups was conducted using a Love Plot (*Fig. S2*).

**Table 1:** Characteristics of patients who underwent colorectal surgery during the study interval

Characteristics	Baseline group	Bundle-1	Bundle-2	*P*
**Colorectal surgery**				
Number of procedures	18 664	3908	9633	
Sex				0.125
Male	11 345	2316	5772	
Female	7319	1592	3861	
Age (years), median (i.q.r.)	69.79 (60.90–78.32)	68.52 (60.97–77.26)	71.08 (61.84–79.05)	<0.001
Duration of intervention (min), median (i.q.r.)	165 (120–218)	164 (125–214)	176 (135–230)	<0.001
Clean-contaminated wound	18 038 (96.6)	3857 (98.7)	9544 (99.1)	<0.001
NISS > = 1	6263 (33.6)	929 (23.8)	2823 (29.3)	<0.001
ASA classification				<0.001
I	1045 (5.6)	221 (5.7)	413 (4.3)	
II	10 333 (55.4)	2260 (57.8)	5241 (54.4)	
III	6837 (36.6)	1378 (35.3)	3804 (39.5)	
IV	449 (2.4)	49 (1.3)	175 (1.8)	
MIS	10 986 (58.9)	2941 (75.3)	7760 (80.6)	<0.001
Adequate i.v. antibiotic prophylaxis	16 266 (87.2)	3187 (81.6)	8218 (85.3)	<0.001
**Colon surgery**				
Number of procedures	13 112	2834	7329	
Sex				0.095
Male	7753	1615	4278	
Female	5359	1219	3051	
Age (years), median (i.q.r.)	70.16 (61.20–78.66)	69.22 (61.62–77.94)	71.71 (62.39–79.27)	<0.001
Duration of intervention (min), median (i.q.r.)	150 (115–195)	151 (120–193)	165 (129–210)	<0.001
Clean-contaminated wound	12 737 (97.1)	2806 (99.0)	7289 (99.5)	<0.001
NISS > = 1	4452 (34.0)	671 (23.7)	2203 (30.1)	<0.001
ASA classification				<0.001
I	734 (5.6)	161 (5.7)	314 (4.3)	
II	7171 (54.7)	1621 (57.2)	3960 (54.0)	
III	4861 (37.1)	1019 (36.0)	2906 (39.7)	
IV	346 (2.6)	33 (1.2)	149 (2.0)	
MIS	7723 (58.9)	2141 (75.5)	5827 (79.5)	<0.001
Adequate i.v. antibiotic prophylaxis	11 476 (87.5)	2322 (81.9)	6254 (85.3)	<0.001
**Rectal surgery**				
Number of procedures	5552	1074	2304	
Sex				0.936
Male	3592	701	1494	
Female	1960	373	812	
Age (years), median (i.q.r.)	68.86 (60.31–77.35)	66.77 (59.18–75.17)	69.43 (60.27–78.13)	<0.001
Duration of intervention (min), median (i.q.r.)	205 (150–265)	205 (160–262)	220 (170–275)	<0.001
Clean-contaminated wound	5301 (95.5)	1051 (97.9)	2255 (97.9)	<0.001
NISS > = 1	1811 (32.6)	258 (24.0)	620 (26.9)	<0.001
ASA classification				0.002
I	311 (5.6)	60 (5.6)	99 (4.3)	
II	3162 (57.0)	639 (59.5)	1281 (55.6)	
III	1976 (35.6)	359 (33.4)	898 (39.0)	
IV	103 (1.9)	16 (1.5)	26 (1.1)	
MIS	3263 (58.8)	800 (74.5)	1933 (83.9)	<0.001
Adequate i.v. antibiotic prophylaxis	4790 (86.3)	865 (80.5)	1964 (85.2)	<0.001

Values are *n* (%) unless otherwise indicated. i.q.r., interquartile range; NNIS, National Nosocomial Infection Surveillance risk index; ASA, American Society of Anesthesiologists surgical risk score; i.v., intravenous; MIS, minimally invasive surgery.

### SSI rates and bundle compliance

**Fig. 1:**
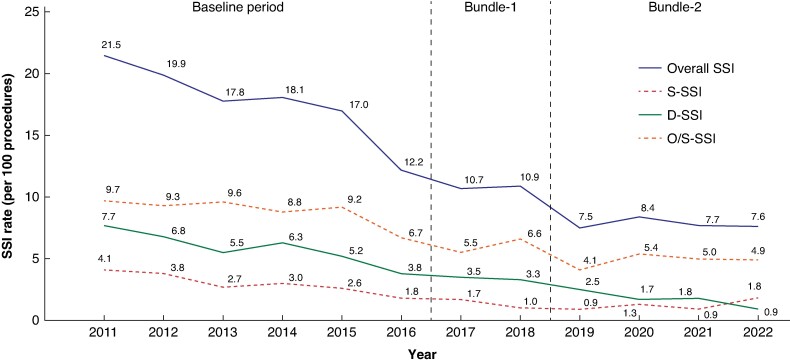
SSI rates throughout the study

SSI, surgical site infection; S-SSI, superficial surgical site infection; D-SSI, deep surgical site infection; O/S-SSI, organ-space surgical site infection.

Overall SSI rate decreased steadily over time: from 18.16% in the Baseline group to 10.03% in the Bundle-1 group and 8.19% in the Bundle-2 group (*Fig. 1*).

In colorectal operations taken together, both bundles significantly decreased overall SSI and its three levels compared with the Baseline group. Specifically, Bundle-2 achieved a 21% reduction in the odds of developing SSI (OR 0.79 (95% c.i. 0.69 to 0.91); *P* = 0.001) along with a 26% decrease in S-SSI (OR 0.74 (95% c.i. 0.58 to 0.95); *P* = 0.018) and a 34% reduction in D-SSI (OR 0.66 (95% c.i. 0.46 to 0.93); *P* = 0.018), compared with Bundle-1. However, Bundle-2 did not show a statistically significant reduction in the likelihood of developing O/S-SSI when compared with Bundle-1 (*Table 2*).

**Table 2:** SSI rates in the three intervals

				Baseline group (ref.) *versus* Bundle-1		Baseline group (ref.) *versus* Bundle-2		Bundle-1 (ref.) *versus* Bundle-2	
SSI	Baseline group	Bundle-1	Bundle-2	OR (95% c.i.)	*P*	OR (95% c.i.)	*P*	OR (95% c.i.)	*P*
**Colorectal surgery**									
Overall-SSI	3390 (18.16)	392 (10.03)	789 (8.19)	0.56 (0.50 to 0.63)	<0.001	0.44 (0.40 to 0.48)	<0.001	0.79 (0.69 to 0.91)	0.001
S-SSI	1119 (6)	125 (3.2)	213 (2.21)	0.59 (0.48 to 0.72)	<0.001	0.44 (0.37 to 0.52)	<0.001	0.74 (0.58 to 0.95)	0.018
D-SSI	569 (3.05)	58 (1.48)	97 (1.01)	0.55 (0.41 to 0.74)	<0.001	0.36 (0.29 to 0.46)	<0.001	0.66 (0.46 to 0.93)	0.018
O/S-SSI	1702 (9.12)	209 (5.35)	479 (4.97)	0.60 (0.51 to 0.70)	<0.001	0.53 (0.47 to 0.59)	<0.001	0.88 (0.74 to 1.06)	0.172
**Colon surgery**									
Overall-SSI	2202 (16.79)	241 (8.5)	512 (6.99)	0.52 (0.45 to 0.60)	<0.001	0.40 (0.36 to 0.45)	<0.001	0.77 (0.65 to 0.92)	0.003
S-SSI	813 (6.2)	81 (2.86)	149 (2.03)	0.52 (0.40 to 0.66)	<0.001	0.39 (0.32 to 0.48)	<0.001	0.76 (0.56 to 1.02)	0.065
D-SSI	305 (2.33)	33 (1.16)	56 (0.76)	0.59 (0.41 to 0.87)	0.007	0.37 (0.27 to 0.50)	<0.001	0.62 (0.39 to 0.99)	0.044
O/S-SSI	1084 (8.27)	127 (4.48)	307 (4.19)	0.56 (0.46 to 0.69)	<0.001	0.48 (0.41 to 0.55)	<0.001	0.85 (0.68 to 1.06)	0.147
**Rectal surgery**									
Overall-SSI	1188 (21.4)	151 (14.06)	277 (12.02)	0.61 (0.50 to 0.74)	<0.001	0.54 (0.46 to 0.63)	<0.001	0.88 (0.70 to 1.11)	0.280
S-SSI	306 (5.51)	44 (4.1)	64 (2.78)	0.79 (0.56 to 1.11)	0.174	0.56 (0.41 to 0.76)	<0.001	0.71 (0.46 to 1.09)	0.121
D-SSI	264 (4.76)	25 (2.33)	41 (1.78)	0.56 (0.36 to 0.87)	0.009	0.41 (0.29 to 0.60)	<0.001	0.74 (0.43 to 1.29)	0.293
O/S-SSI	618 (11.13)	82 (7.64)	172 (7.47)	0.62 (0.48 to 0.80)	<0.001	0.65 (0.54 to 0.79)	<0.001	1.06 (0.79 to 1.42)	0.706

Values are *n* (%) unless otherwise stated. OR, odds ratio; SSI, surgical site infection; S-SSI, superficial surgical site infection; D-SSI, deep surgical site infection; O/S-SSI, organ-space surgical site infection.

Assessing colonic and rectal operations separately: significant reductions in SSI were noted with the application of Bundle-1 and Bundle-2. In colonic procedures, Bundle-2 achieved a 23% reduction in the odds of overall SSI (OR 0.77 (95% c.i. 0.65 to 0.92); *P* = 0.003) and a significant 38% reduction in D-SSI (OR 0.62 (95% c.i. 0.39 to 0.99); *P* = 0.044) compared with Bundle-1. However, Bundle-2 did not show statistically significant differences in the odds of developing S-SSI and O/S-SSI compared with Bundle-1. In rectal operations, significant reductions were observed in overall SSI, S-SSI, and D-SSI with Bundle-1 and Bundle-2. However, Bundle-2 did not confer additional benefits in reducing any of the SSI categories, presenting only non-significant differences compared with Bundle-1.

### Individual effect of bundle measures on SSI rates

In the univariable analysis of colorectal procedures considered together (*Table 3*), all measures, except adequate antibiotic prophylaxis in rectal surgery, reduced SSI. Multivariable analysis demonstrated that laparoscopy, OAP, use of double-ring wound retractor and skin antisepsis with CHG-alcohol decreased SSI. Similar results were observed in colon surgery, while in the rectum, OAP did not reduce SSI.

**Table 3:** Effect of the individual preventive measures contained in the bundles on overall SSI rates

	Univariate		Multivariate	
Bundle measures	OR (95% c.i.)	*P*	OR (95% c.i.)	*P*
**Colorectal surgery**				
Adequate antibiotic prophylaxis	0.90 (0.83 to 0.98)	0.012	0.91 (0.83 to 0.99)	0.022
Minimally invasive surgery	0.53 (0.49 to 0.56)	<0.001	0.62 (0.58 to 0.66)	<0.001
Oral antibiotic prophylaxis	0.41 (0.38 to 0.44)	<0.001	0.68 (0.59 to 0.79)	<0.001
Mechanical bowel preparation	0.44 (0.40 to 0.47)	<0.001	0.92 (0.80 to 1.06)	0.237
Double-ring wound retractor	0.41 (0.38 to 0.45)	<0.001	0.70 (0.63 to 0.79)	<0.001
Maintenance of normothermia	0.45 (0.42 to 0.48)	<0.001	0.95 (0.82 to 1.08)	0.430
Adequate hair removal	0.54 (0.48 to 0.61)	<0.001	1.14 (0.99 to 1.31)	0.074
2% chlorhexidine in alcohol	0.43 (0.39 to 0.47)	<0.001	0.75 (0.65 to 0.86)	<0.001
Glycaemic control	0.47 (0.43 to 0.52)	<0.001	0.95 (0.83 to 1.08)	0.418
Changing of surgical instruments	0.58 (0.42 to 0.77)	<0.001	1.20 (0.87 to 1.63)	0.243
**Colon surgery**				
Adequate antibiotic prophylaxis	0.88 (0.80 to 0.98)	0.018	0.88 (0.80 to 0.98)	0.021
Minimally invasive surgery	0.48 (0.44 to 0.51)	<0.001	0.56 (0.52 to 0.61)	<0.001
Oral antibiotic prophylaxis	0.34 (0.31 to 0.38)	<0.001	0.58 (0.49 to 0.68)	<0.001
Mechanical bowel preparation	0.38 (0.34 to 0.42)	<0.001	0.85 (0.72 to 1.01)	0.061
Double-ring wound retractor	0.40 (0.36 to 0.44)	<0.001	0.79 (0.68 to 0.91)	0.002
Maintenance of normothermia	0.43 (0.39 to 0.47)	<0.001	0.96 (0.81 to 1.13)	0.606
Adequate hair removal	0.51 (0.44 to 0.59)	<0.001	1.07 (0.90 to 1.27)	0.446
2% chlorhexidine in alcohol	0.42 (0.38 to 0.47)	<0.001	0.80 (0.67 to 0.95)	0.011
Glycaemic control	0.45 (0.40 to 0.51)	<0.001	0.92 (0.78 to 1.09)	0.336
Changing of surgical instruments	0.62 (0.43 to 0.86)	0.006	1.28 (0.88 to 1.81)	0.172
**Rectal surgery**				
Adequate antibiotic prophylaxis	0.95 (0.82 to 1.09)	0.449	0.95 (0.83 to 1.11)	0.532
Minimally invasive surgery	0.63 (0.57 to 0.71)	<0.001	0.72 (0.64 to 0.81)	<0.001
Oral antibiotic prophylaxis	0.55 (0.48 to 0.62)	<0.001	0.96 (0.73 to 1.27)	0.757
Mechanical bowel preparation	0.53 (0.47 to 0.61)	<0.001	0.83 (0.63 to 1.10)	0.191
Double-ring wound retractor	0.49 (0.42 to 0.57)	<0.001	0.72 (0.59 to 0.88)	0.002
Maintenance of normothermia	0.53 (0.46 to 0.60)	<0.001	0.88 (0.70 to 1.12)	0.310
Adequate hair removal	0.63 (0.52 to 0.77)	<0.001	1.18 (0.93 to 1.49)	0.181
2% chlorhexidine in alcohol	0.50 (0.43 to 0.59)	<0.001	0.76 (0.61 to 0.96)	0.020
Glycaemic control	0.56 (0.47 to 0.67)	<0.001	0.99 (0.79 to 1.25)	0.962
Changing of surgical instruments	0.55 (0.28 to 0.98)	0.062	0.97 (0.48 to 1.78)	0.931

OR, odds ratio; SSI, surgical site infection.

For O/S-SSI, laparoscopy, OAP, MBP, double-ring wound retractor and CHG-alcohol were protective factors in colorectal surgery (*Table 4*). In colonic operations, laparoscopy, OAP and MBP protected from O/S-SSI, but no efficacy was identified for any measure in rectal surgery.

**Table 4:** Effect of the individual preventive measures contained in the bundles on O/S-SSI rates

	Univariate		Multivariate	
Bundle measures	OR (95% c.i.)	*P*	OR (95% c.i.)	*P*
**Colorectal surgery**				
Adequate antibiotic prophylaxis	0.89 (0.80 to 1.00)	0.046	0.90 (0.81 to 1.01)	0.061
Minimally invasive surgery	0.69 (0.63 to 0.75)	<0.001	0.78 (0.72 to 0.85)	<0.001
Oral antibiotic prophylaxis	0.53 (0.47 to 0.58)	<0.001	0.82 (0.68 to 0.98)	0.029
Mechanical bowel preparation	0.53 (0.48 to 0.59)	<0.001	0.82 (0.69 to 0.99)	0.036
Double-ring wound retractor	0.54 (0.49 to 0.60)	<0.001	0.82 (0.71 to 0.95)	0.010
Maintenance of normothermia	0.56 (0.51 to 0.61)	<0.001	0.98 (0.82 to 1.16)	0.789
Adequate hair removal	0.65 (0.56 to 0.75)	<0.001	1.13 (0.94 to 1.35)	0.177
2% chlorhexidine in alcohol	0.53 (0.48 to 0.60)	<0.001	0.78 (0.66 to 0.93)	0.006
Glycaemic control	0.58 (0.51 to 0.65)	<0.001	0.94 (0.79 to 1.12)	0.501
Changing of surgical instruments	0.61 (0.40 to 0.89)	0.016	1.00 (0.64 to 1.48)	0.992
**Colon surgery**				
Adequate antibiotic prophylaxis	0.88 (0.76 to 1.01)	0.062	0.88 (0.77 to 1.01)	0.067
Minimally invasive surgery	0.60 (0.54 to 0.67)	<0.001	0.69 (0.62 to 0.77)	<0.001
Oral antibiotic prophylaxis	0.46 (0.40 to 0.53)	<0.001	0.74 (0.60 to 0.93)	0.008
Mechanical bowel preparation	0.46 (0.40 to 0.52)	<0.001	0.73 (0.59 to 0.91)	0.005
Double-ring wound retractor	0.53 (0.47 to 0.60)	<0.001	0.94 (0.78 to 1.14)	0.516
Maintenance of normothermia	0.53 (0.47 to 0.60)	<0.001	0.96 (0.77 to 1.20)	0.749
Adequate hair removal	0.61 (0.51 to 0.73)	<0.001	1.07 (0.86 to 1.34)	0.536
2% chlorhexidine in alcohol	0.52 (0.45 to 0.60)	<0.001	0.85 (0.68 to 1.07)	0.164
Glycaemic control	0.54 (0.46 to 0.63)	<0.001	0.86 (0.69 to 1.07)	0.178
Changing of surgical instruments	0.76 (0.48 to 1.14)	0.217	1.26 (0.78 to 1.93)	0.313
**Rectal surgery**				
Adequate antibiotic prophylaxis	0.94 (0.78 to 1.14)	0.518	0.95 (0.79 to 1.15)	0.574
Minimally invasive surgery	0.87 (0.76 to 1.01)	0.066	0.96 (0.83 to 1.12)	0.624
Oral antibiotic prophylaxis	0.66 (0.55 to 0.77)	<0.001	0.95 (0.67 to 1.35)	0.768
Mechanical bowel preparation	0.65 (0.55 to 0.76)	<0.001	0.82 (0.57 to 1.16)	0.268
Double-ring wound retractor	0.63 (0.52 to 0.76)	<0.001	0.84 (0.66 to 1.09)	0.189
Maintenance of normothermia	0.66 (0.56 to 0.77)	<0.001	0.95 (0.70 to 1.28)	0.729
Adequate hair removal	0.77 (0.60 to 0.97)	0.031	1.14 (0.85 to 1.53)	0.378
2% chlorhexidine in alcohol	0.62 (0.51 to 0.76)	<0.001	0.77 (0.58 to 1.02)	0.069
Glycaemic control	0.72 (0.58 to 0.88)	0.002	1.10 (0.83 to 1.47)	0.498
Changing of surgical instruments	0.28 (0.07 to 0.75)	0.030	0.39 (0.09 to 1.05)	0.110

OR, odds ratio; O/S-SSI, organ-space surgical site infection.

For I-SSI, laparoscopy, OAP, double-ring wound retractor and CHG-alcohol were preventive measures in colorectal and colonic procedures (*Table S3*), while in rectal operations only laparoscopy and wound retractor were independent protective factors.

### Secondary outcomes

**Fig. 2:**
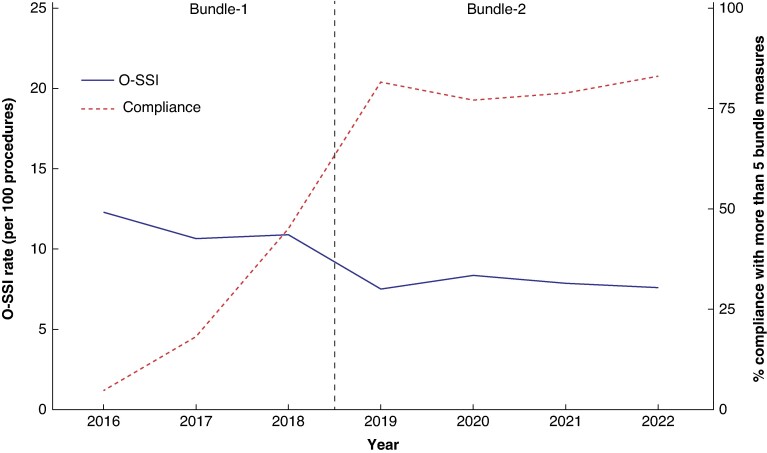
Relationship between the percentage of compliance with five or more bundle measures and overall SSI rate

All measures included in both bundles were adopted with an average adherence rate of 70–80%, which increased over time (*Table S4*). Compliance with five or more measures increased over the course of the study and was associated with a reduction in the SSI rate (*Fig. 2*).

O-SSI, overall surgical site infection.

LOS fell significantly over time, from 7 in the Baseline group to 6 and 5 days for Bundle-1 and Bundle-2 respectively (*P* < 0.001) (*Table 5*). There was no difference in the median time to SSI development in the study interval, while more SSIs were diagnosed after discharge (from 29 to 40%) with the application of bundles.

**Table 5:** Secondary outcomes of the study

				Baseline group (ref.) *versus* Bundle-1		Baseline group (ref.) *versus* Bundle-2		Bundle-1 (ref.) *versus* Bundle-2	
Outcomes	Baseline group	Bundle-1	Bundle-2		*P*		*P*		*P*
**Colorectal surgery**									
LOS (days), median (i.q.r.)*	7 (5–11)	6 (4–8)	5 (4–8)	1	<0.001	2	<0.001	1	<0.001
Days to SSI, median (i.q.r.)*	7 (5–12)	8 (5–13)	8 (4–13.5)	1	0.180	1	0.466	0	0.561
30-day mortality rate, *n* (%)†	261 (1.4)	23 (0.59)	58 (0.6)	0.53 (0.34 to 0.83)	0.005	0.51 (0.37 to 0.71)	<0.001	0.97 (0.58 to 1.64)	0.916
Postdischarge SSI, *n* (%)†	937 (29.53)	105 (35.23)	138 (40.47)	1.24 (0.96 to 1.62)	0.105	1.54 (1.2 to 1.97)	<0.001	1.24 (0.88 to 1.74)	0.227
**Colon surgery**									
LOS (days), median (i.q.r.)*	7 (5–10)	6 (4–8)	5 (4–7)	1	<0.001	2	<0.001	1	<0.001
Days to SSI, median (i.q.r.)*	7 (5–11)	7 (5–12)	7 (4–12)	0	0.178	0	0.234	0	0.745
30-day mortality rate, *n* (%)†	203 (1.55)	18 (0.64)	50 (0.68)	0.55 (0.33 to 0.91)	0.019	0.56 (0.39 to 0.8)	0.001	1.02 (0.57 to 1.82)	0.946
Postdischarge SSI, *n* (%)†	592 (28.82)	69 (36.7)	82 (39.23)	1.34 (0.96 to 1.85)	0.082	1.49 (1.08 to 2.04)	0.014	1.11 (0.72 to 1.72)	0.63
**Rectal Surgery**									
LOS (days), median (i.q.r.)*	8 (6–13)	6 (5–10)	6 (4–9)	2	<0.001	2	<0.001	0	<0.001
Days to SSI, median (i.q.r.)*	9 (5–14)	9 (5–14.5)	9 (5–16)	0	0.815	0	0.798	0	0.758
30-day mortality rate, *n* (%)†	58 (1.04)	5 (0.47)	8 (0.35)	0.46 (0.17 to 1.24)	0.123	0.28 (0.13 to 0.59)	<0.001	0.6 (0.18 to 1.98)	0.402
Postdischarge SSI, *n* (%)†	345 (30.83)	36 (32.73)	56 (42.42)	1.1 (0.7 to 1.72)	0.69	1.67 (1.11 to 2.52)	0.014	1.53 (0.85 to 2.73)	0.154

*Absolute difference in medians; †OR (95% c.i.) in comparative columns. i.q.r., interquartile range; LOS, duration of stay; SSI, surgical site infection.

The mortality rate fell over time, from 1.4% to 0.59% and 0.6% at Baseline, Bundle-1 and Bundle-2 (*P* < 0.001) respectively. Measures added in Bundle-2 did not reduce the mortality rate.

The microorganisms causing SSI in colorectal surgery were variable throughout the study. In the O/S-SSI category (*Table 6*), Bundle-1 led to a higher isolation of Gram-positive bacteria (mainly due to an increase of *Enterococcus faecalis* and *Enterococcus faecium*), and yeasts (*Candida* spp.), and decreased Gram-negative bacteria, specifically *Escherichia coli, Pseudomonas* spp. and anaerobes (*Bacteroides* spp.), compared with the Baseline group. Bundle-2 added some benefits to Bundle-1, reducing *Bacteroides* spp. Microorganisms causing I-SSI did not change with Bundle-1 in comparison to Baseline (*Table 6*). However, Bundle-2 was associated with an increase in Gram-positive (mainly methicillin-susceptible *Staphylococcus aureus*) and a reduction in Gram-negative bacteria (*Escherichia coli*) and anaerobes.

**Table 6:** Microorganisms causing O/S-SSI and I-SSI throughout the study

Organisms isolated in O/S-SSI					*P*		
Organisms	Overall	Baseline group	Bundle-1	Bundle-2	Baseline group (ref.) *versus* Bundle 1	Baseline group (ref.) *versus* Bundle 2	Bundle-1 (ref.) *versus* Bundle 2
Number	2910	2117	245	548			
**Gram-positive bacteria**	632 (21.7)	413 (19.5)	75 (30.6)	144 (26.3)	<0.001	0.001	0.229
*Enterococcus faecalis*	259 (8.9)	164 (7.7)	34 (13.9)	61 (11.1)	0.002	0.015	0.288
*Enterococcus faecium*	234 (8.0)	151 (7.1)	28 (11.4)	55 (10.0)	0.021	0.031	0.616
*Enterococcus* spp.	33 (1.1)	27 (1.3)	2 (0.8)	4 (0.7)	0.762	0.374	1.000
Streptococcus spp.	34 (1.2)	25 (1.2)	2 (0.8)	7 (1.3)	1.000	0.827	0.729
MRSA	13 (0.4)	9 (0.4)	2 (0.8)	2 (0.4)	0.319	1.000	0.591
MSSA	29 (1.0)	20 (0.9)	5 (2.0)	4 (0.7)	0.173	0.802	0.144
Other GPB	93 (3.2)	62 (2.9)	9 (3.7)	22 (4.0)	0.551	0.216	1.000
**Gram-negative bacteria**	1361 (46.8)	1030 (48.7)	106 (43.3)	225 (41.1)	0.120	0.002	0.586
*Escherichia coli*	714 (24.5)	567 (26.8)	54 (22.0)	93 (17.0)	0.125	<0.001	0.093
*Klebsiella* spp.	5 (0.2)	1 (0.0)	1 (0.4)	3 (0.5)	0.197	0.029	1.000
*Pseudomonas* spp.	161 (5.5)	128 (6.0)	5 (2.0)	28 (5.1)	0.008	0.475	0.053
*Enterobacter* spp.	13 (0.4)	9 (0.4)	1 (0.4)	3 (0.5)	1.000	0.720	1.000
Other GNB	468 (16.1)	325 (15.4)	45 (18.4)	98 (17.9)	0.227	0.149	0.920
**Anaerobes**	94 (3.2)	77 (3.6)	9 (3.7)	8 (1.5)	1.000	0.009	0.062
*Bacteroides* spp.	74 (2.5)	62 (2.9)	8 (3.3)	4 (0.7)	0.693	0.002	0.011
*Clostridium* spp.	16 (0.5)	13 (0.6)	1 (0.4)	2 (0.4)	1.000	0.750	1.000
**Yeasts**	103 (3.5)	54 (2.6)	9 (3.7)	40 (7.3)	0.294	<0.001	0.055
*Candida* spp.	103 (3.5)	54 (2.6)	9 (3.7)	40 (7.3)	0.294	<0.001	0.055

Organisms isolated in incisional-SSI					*P*		
Organisms	Overall	Baseline group	Bundle-1	Bundle-2	Baseline group (ref.) *versus* Bundle 1	Baseline group (ref.) *versus* Bundle 2	Bundle-1 (ref.) *versus* Bundle 2
Number	2538	1979	217	342			
**Gram-positive bacteria**	536 (21.1)	390 (19.7)	49 (22.6)	97 (28.4)	0.325	<0.001	0.139
*Enterococcus faecalis*	214 (8.4)	159 (8.0)	24 (11.1)	31 (9.1)	0.153	0.522	0.468
*Enterococcus faecium*	76 (3.0)	53 (2.7)	6 (2.8)	17 (5.0)	0.827	0.038	0.275
*Enterococcus* spp.	19 (0.7)	18 (0.9)	1 (0.5)	0 (0.0)	1.000	0.094	0.388
Streptococcus spp.	42 (1.7)	38 (1.9)	0 (0.0)	4 (1.2)	0.028	0.508	0.161
MRSA	20 (0.8)	15 (0.8)	1 (0.5)	4 (1.2)	1.000	0.509	0.653
MSSA	106 (4.2)	64 (3.2)	14 (6.5)	28 (8.2)	0.031	<0.001	0.512
Other GPB	207 (8.2)	145 (7.3)	17 (7.8)	45 (13.2)	0.784	0.001	0.054
**Gram-negative bacteria**	1327 (52.3)	1051 (53.1)	118 (54.4)	158 (46.2)	0.774	0.019	0.068
*Escherichia coli*	720 (28.4)	596 (30.1)	53 (24.4)	71 (20.8)	0.085	<0.001	0.347
*Klebsiella* spp.	1 (0.0)	0 (0.0)	0 (0.0)	1 (0.3)	1.000	0.147	1.000
*Pseudomonas* spp.	176 (6.9)	129 (6.5)	22 (10.1)	25 (7.3)	0.064	0.558	0.274
*Enterobacter* spp.	8 (0.3)	7 (0.4)	0 (0.0)	1 (0.3)	1.000	1.000	1.000
Other GNB	422 (16.6)	319 (16.1)	43 (19.8)	60 (17.5)	0.177	0.526	0.504
**Anaerobes**	98 (3.9)	85 (4.3)	8 (3.7)	5 (1.5)	0.859	0.009	0.147
*Bacteroides* spp.	93 (3.7)	82 (4.1)	6 (2.8)	5 (1.5)	0.464	0.013	0.352
*Clostridium* spp.	4 (0.2)	3 (0.2)	1 (0.5)	0 (0.0)	0.341	1.000	0.388
**Yeasts**	22 (0.9)	17 (0.9)	1 (0.5)	4 (1.2)	1.000	0.537	0.653
*Candida* spp.	22 (0.9)	17 (0.9)	1 (0.5)	4 (1.2)	1.000	0.537	0.653

Values are *n* (%) unless otherwise indicated. O/S-SSI, organ/space-surgical site infection; MRSA, methicillin-resistant *Staphylococcus aureus*; MSSA, methicillin-sensitive *Staphylococcus aureus;* GPB, Gram-positive bacteria; GNB, Gram-negative bacteria.

## Discussion

This prospective multicentre cohort study demonstrated a 50% reduction in overall SSI after the implementation of two sequential sets of bundles, from 18.16% to 8.19%. These results were recorded by different hospitals and ICTs, although it is important to note that this was probably made possible by leveraging a well established nationwide surveillance system for healthcare-associated infections. Similar results have been reported previously^9,12,26–28^.

The addition of new measures (from a six-measure protocol in Bundle-1 to a 10-measure protocol in Bundle-2) within a national SSI surveillance programme increased compliance. Adherence above 70% is favourable compared with previous studies reporting adherence rates between 50 and 70%. These two packages were successfully introduced in less than a year, building on a well established national surveillance system for health-related infections in a large network of hospitals. The application of bundles in similar multicentre collaborative settings has shown that quality improvement projects can be easier to implement in these environments^29^.

Although bundles with a large number of measures may face greater challenges in terms of implementation^30^, this study corroborates two previous meta-analyses^9,17^ in demonstrating that bundles that include 10 or more measures implemented correctly can lead to the greatest reduction in SSI. In order to achieve this, involvement of stakeholders in its implementation is crucial, giving feedback on the results and taking advantage of their new ideas to improve compliance^30^.

Application of Bundle-1 and Bundle-2 reduced not only incisional SSI but O/S-SSI as well. Previously published colorectal bundles have been found to be effective in reducing I-SSI but did not improve O/S-SSI^12,28^. These results are relevant because the effects of O/S-SSI are more impactful compared with I-SSI in terms of LOS, 30-day mortality rate (from 2% without O/S-SSI to 24% with it)^31^ and reducing long-term survival^32^.

However, in this cohort the effect of the bundles was different for I-SSIs and O/S-SSIs: the addition of the four extra measures in Bundle-2 only reduced I-SSIs and did not significantly influence O/S-SSIs. These differences may be explained as Bundle-1 measures were specifically chosen to reduce not just overall SSI and O/S-SSIs in colorectal surgery, whereas the Bundle-2 measures were added in the surveillance programme with the aim of reducing the SSI rate in all types of surgical procedures and were perhaps more targeted at I-SSIs. O/S-SSI has traditionally been related to anastomotic leakage, which is assumed to be related to technical factors in the construction of the anastomosis, such as ensuring a good blood supply and the absence of tension, and the creation of a protective stoma in high-risk groups^33,34^. In addition, recent research has highlighted other aspects such as the diversity and composition of the colonic microbiota or intraoperative resuscitation as contributing factors^35–37^. Several studies demonstrated in animal models that alteration of the gut microbiome involving the growth of specific microorganisms, such as *Pseudomonas aeruginosa* and *Enterococcus* spp., could lead to tissue destruction and anastomotic leakage^38,39^.

In contrast to most studies, this study analysed colon and rectal surgery separately as these two types of surgery have different SSI risk factors, intraoperative technical factors and postoperative management^40,41^. Although the bundles reduced SSI overall, when analysing the individual effect of the items included, the multivariable study detected differences between colon and rectal surgery. In colonic surgery, the protective factors for I-SSI were the use of laparoscopy, OAP, double-ring wound retractor and CHG-alcohol skin antisepsis, while in rectal surgery only laparoscopy and double-ring wound retractor were significant.

In cases of O/S-SSI, laparoscopy, OAP and MBP were beneficial in colon surgery, but none of the factors were protective in rectal surgery. Notably, no single protective factor was found for O/S-SSI in rectal surgery. The more demanding technical aspects of this surgery, such as the potential need for neoadjuvant radiotherapy, the proximity of the sphincters, the high-risk distal anastomoses and the narrow pelvis may counteract the positive effect of the measures included in the bundles^42–44^. A study of patients analysing risk factors and outcomes of O/S-SSI after elective colon and rectal surgery showed that the overall O/S-SSI rates were higher in rectal surgery. Patients were younger but had a higher proportion of malignancy, received chemoradiotherapy more frequently and had a longer duration of surgery. Surgical techniques were also different, with a higher proportion of patients requiring stomas^45^.

As for the maintenance of normothermia, the hypothesis was that this would achieve better intraoperative homeostasis, as previously demonstrated with other haemodynamic parameters^36^. This would reduce anastomotic leakage and, in turn, O/S-SSI, but this was not demonstrated as a protective effect. This apparent lack of any benefit in maintaining normothermia can be attributed to the fact that the difference in temperature between the SSI and non-SSI patient groups was only 0.1°C. As all patients are currently undergoing perioperative warming, the temperature differences are marginal and do not reach statistical significance as a preventive measure of O/S-SSI.

It is clear that during the study interval there have been advances in care practices that may have acted as confounding factors in the evaluation of the particular interventions applied by the programme. The most important of these is the widespread introduction of the laparoscopic technique in colorectal surgery. Laparoscopy has been shown to reduce overall and incisional SSIs, although most studies find no effect on O/S-SSIs^46,47^. Instead, in this series, the stepwise introduction of laparoscopy acted as a significant preventive factor not only for general and incisional SSIs, but also for O/S-SSIs, although to a lesser extent.

Compliance with well founded evidence-based measures and the fall in SSI are associated with improvements in LOS and the mortality rate (from 1.4% to 0.6%). As a result of shorter LOS, more SSIs were detected after discharge, a circumstance that should encourage the design of methods to detect infectious complications before discharge, especially O/S-SSI. Several studies have included C-reactive protein (CRP) as a guide for early detection of anastomotic leaks. This assessment if properly applied in Enhance Recovery After Surgery protocols is important for early and safe patient discharge^48–53^. Three meta-analyses concluded that with CRP levels below 150 mg/l on postoperative day 3, anastomotic leakage can be ruled out in 97% of patients^54–56^. In addition, it has recently been shown that a CRP-based protocol in elective colorectal surgery provides better results in terms of anastomotic salvage^57^.

Although we found OAP to be successful in reducing SSIs, its use has probably led to a change in the microorganisms isolated from SSIs after colorectal surgery. There was a significant reduction in Gram-negative bacteria but an increase in Gram-positive bacteria, mainly *Enterococcus* spp., with a substantial increase in *E. faecium* and yeasts in accordance with previous studies^58–60^. In experimental animal studies, oral antibiotics (for example neomycin) changed the diversity of the gut microbiota and increased the presence of potentially pathogenic genera such as *Enterococcus*^54^. This information should be considered when elderly patients with significant morbidities develop severe SSI after colorectal surgery; in these cases, perhaps empirical antibiotic treatment covering these aetiologies should be considered.

This study has several limitations. First, as this is based on population-based databases, information on other factors that might influence the occurrence of SSI, such as body mass index, smoking and co-morbidities, or on surgical factors such as the type of anastomosis or the occurrence of anastomotic leakage, was not available. It is also possible that some of the recommendations introduced later in the bundles, such as changing instruments before wound closure, for example, were already implemented at some participating hospitals, but it cannot be established which ones, or to what extent. Additionally, over the long time interval analysed in the study there have been changes in clinical practice (for instance, the increasing use of laparoscopy) which may have influenced the results.

## Collaborators

### VINCat Colorectal Surveillance Team

Domenico Fraccalvieri (Department of Surgery, Hospital Universitari de Bellvitge, L'Hospitalet de Llobregat, Spain); Ana Abad-Torrent (Department of Anaesthesiology, Hospital Universitari Vall d'Hebrón, Barcelona, Spain); Alejandro Solís-Peña (Department of Surgery, Hospital Universitari Vall d'Hebrón, Barcelona, Spain); Mireia Puig-Asensio (Department of Infectious Diseases, Hospital Universitari de Bellvitge, L'Hospitalet de Llobregat, Spain); Lucrecia López (Infection Control Team, Hospital de Moisès Broggi, Sant Joan Despí, Spain); Marta Piriz (Infection Control Team, Hospital Universitari Sant Pau, Barcelona, Spain); Mercè Hernández (Department of Surgery, Hospital Universitari Parc Taulí, Sabadell, Spain); Dolors Castellana (Hospital Universitari Arnau de Vilanova, Lleida, Spain); Elisa Montiu González (Hospital Universitari Arnau de Vilanova, Lleida, Spain); Graciano García Pardo (Hospital Universitari Joan XXIII, Tarragona, Spain); Francesc Feliu Villaró (Hospital Universitari Joan XXIII, Tarragona, Spain); Josep Rebull Fatsini (Hospital Verge de la Cinta, Tortosa, Spain); Marie France Domènech Spaneda (Hospital Verge de la Cinta, Tortosa, Spain); Marta Conde Galí (Hospital Universitari Dr. Josep Trueta, Girona, Spain); Anna Oller Pérez-Hita (Hospital Universitari Dr. Josep Trueta, Girona, Spain); Lydia Martín (Hospital de Viladecans, Viladecans, Spain); Ana Lerida (Hospital de Viladecans, Viladecans, Spain); Sebastiano Biondo (Hospital Universitari de Bellvitge, L'Hospitalet de LLobregat, Spain); Emilio Jiménez Martínez (Hospital Universitari de Bellvitge, L'Hospitalet de LLobregat, Spain); Nieves Sopena Galindo (Hospital Universitari Germans Tries i Pujol, Badalona, Spain); Ignasi Camps Ausàs (Hospital Universitari Germans Tries i Pujol, Badalona, Spain); Carmen Ferrer (Hospital Universitari Vall d'Hebron, Barcelona, Spain); Luis Salas (Hospital Universitari Vall d'Hebron, Barcelona, Spain); Rafael Pérez Vidal (Althaia Xarxa Assistencial, Manresa, Spain); Dolors Mas Rubio (Althaia Xarxa Assistencial, Manresa, Spain); Irene García de la Red (Hospital HM Delfos, Barcelona, Spain); Mª Angels Iruela Castillo (Clínica Girona, Girona, Spain); Eva Palau i Gil (Clínica Girona, Girona, Spain); José Antonio Martínez (Hospital Clínic de Barcelona, Barcelona, Spain); M. Blanca Torralbo Navarro (Hospital Clínic de Barcelona, Barcelona, Spain); Maria López (Hospital Universitari Mútua de Terrassa, Terrassa, Spain); Carol Porta (Hospital Universitari Mútua de Terrassa, Terrassa, Spain); Alex Smithson Amat (Fundació Hospital de l'Esperit Sant, Santa Coloma de Gramenet, Spain); Guillen Vidal Escudero (Fundació Hospital de l'Esperit Sant, Santa Coloma de Gramenet, Spain); José Carlos de la Fuente Redondo (Hospital Comarcal Mora d'Ebre, Mora d'Ebre, Spain); Montse Rovira Espés (Hospital Comarcal Mora d'Ebre, Mora d'Ebre, Spain); Arantxa Mera Fidalgo (Hospital de Palamós, Palamós, Spain); Luis Escudero Almazán (Hospital de Palamós, Palamós, Spain); Monserrat Ortega Raya (Hospital Parc Taulí de Sabadell, Sabadell, Spain); Mª Carmen Álvarez Moya (Parc Sanitari Sant Joan de Déu, Sant Boi, Spain); Vicens Diaz-Brito (Parc Sanitari Sant Joan de Déu, Sant Boi, Spain); Laura Grau Palafox (Hospital de Terrassa, Terrassa, Spain); Yésika Angulo Gómez (Hospital de Terrassa, Terrassa, Spain); Anna Besolí Codina (Consorci Hospitalari de Vic, Vic, Spain); Carme Autet Ricard (Consorci Hospitalari de Vic, Vic, Spain); Carlota Hidalgo López (Hospital del Mar, Barcelona, Spain); Elisabeth Lerma-Chippirraz (Hospital General de Granollers, Granollers, Spain); Demelza Maldonado López (Hospital General de Granollers, Granollers, Spain); David Blancas (Consorci Sanitari del Garraf, Vilanova i la Geltrú, Spain); Esther Moreno Rubio (Consorci Sanitari del Garraf, Vilanova i la Geltrú, Spain); Roser Ferrer i Aguilera (Hospital Sant Jaume de Calella, Calella, Spain); Simona Iftimie (Hospital Universitari Sant Joan de Reus, Reus, Spain); Antoni Castro-Salomó (Hospital Universitari Sant Joan de Reus, Reus, Spain); Rosa Laplace Enguídanos (Hospital de Sant Pau i Santa Tecla, Tarragona, Spain); Maria Carmen Sabidó Serra (Hospital de Sant Pau i Santa Tecla, Tarragona, Spain); Núria Bosch Ros (Hospital de Santa Caterina, Salt, Spain); Virginia Pomar Solchaga (Hospital de la Santa Creu i Sant Pau, Barcelona, Spain); Laura Lázaro Garcia (Hospital Universitari Quirón Dexeus, Barcelona, Spain); Angeles Boleko Ribas (Hospital Universitari Quirón Dexeus, Barcelona, Spain); Jordi Palacín Luque (Pius Hospital de Valls, Valls, Spain); Alexandra Lucía Moise (Pius Hospital de Valls, Valls, Spain); Mª Carmen Fernández Palomares (Hospital Universitari Sagrat Cor, Barcelona, Spain); Santiago Barba Sopeña (Hospital Universitari Sagrat Cor, Barcelona, Spain); Eduardo Sáez Huertas (Clínica Nova Aliança, Lleida, Spain); Sara Burges Estada (Clínica Nova Aliança, Lleida, Spain); Josep María Tricas Leris (Fundació privada Hospital de Mollet, Mollet, Spain); Eva Redon Ruiz (Fundació privada Hospital de Mollet, Mollet, Spain); Montse Brugués (Consorci Sanitari de l'Anoia, Igualada, Spain); Susana Otero Aced (Consorci Sanitari de l'Anoia, Igualada, Spain); Maria Cuscó Esteve (Hospital Comarcal de l'Alt Penedès, Vilafranca del Penedés, Spain); Francisco José Vargas-Machuca (Centre MQ de Reus, Reus, Spain); Mª de Gracia García Ramírez (Centre MQ de Reus, Reus, Spain); Ana Maria Ciscar Bellés (Consorci Hospitalari del Maresme, Mataró, Spain); Elena Vidal Díez (Consorci Hospitalari del Maresme, Mataró, Spain); Mariló Marimón Morón (Hospital Universitari General de Catalunya, Sant Cugat, Spain); Marisol Martínez Sáez (Hospital Universitari General de Catalunya, Sant Cugat, Spain); Josep Farguell (QUIRON Salud, Barcelona, Spain); Mireia Saballs (QUIRON Salud, Barcelona, Spain); Montserrat Vaqué Franco (Hospital de Barcelona, Barcelona, Spain); Leonor Invernón Garcia (Hospital de Barcelona, Barcelona, Spain); Rosa Laplace Enguídanos (Hospital Comarcal del Vendrell, El Vendrell, Spain); Meritxell Guillemat Marrugat (Hospital Comarcal del Vendrell, El Vendrell, Spain); Ana Coloma Conde (Hospital Moisès Broggi, Sant Joan Despí, Spain).

## Supplementary Material

zrae080_Supplementary_Data

## Data Availability

The research data is prospectively registered and belongs to the Surveillance of Healthcare Related Infections in Catalonia Program (VINCat), a program from the Catalan Health Service, Department of Health, Generalitat de Catalunya. All data will be made available on request.
