# Correction to: Comparison of two bundles for reducing surgical site infection in colorectal surgery: multicentre cohort study

**DOI:** 10.1093/bjsopen/zrag009

**Published:** 2026-03-05

This is a correction to: Miriam Flores-Yelamos, Aina Gomila-Grange, Josep M Badia, Alexander Almendral, Ana Vázquez, David Parés, Marta Pascual, Enric Limón, Miquel Pujol, Montserrat Juvany, members of the VINCat Colorectal Surveillance Team, Comparison of two bundles for reducing surgical site infection in colorectal surgery: multicentre cohort study, *BJS Open*, Volume 8, Issue 4, August 2024, zrae080, https://doi.org/10.1093/bjsopen/zrae080

The following should be added to the **Funding** statement in the article: “Fundación Privada Hospital Asil de Granollers is a beneficiary of Acción Estratégica en Salud grants funded by Instituto de Salud Carlos III and co-funded by the European Union through the European Regional Development Fund. Project PI19/01294: Profilaxis de la infección de localización quirúrgica incisional con solución antibiótica tópica. A way of building Europe.”

This emendation has been outlined only in this correction notice to preserve the version of record.

