# Supplementary material for: Comparison of two bundles for reducing surgical site infection in colorectal surgery: multicentre cohort study
Source: BJS Open. 2024 Aug 6;8(4):zrae080. doi: 10.1093/bjsopen/zrae080 (PMC11303006; doi:10.1093/bjsopen/zrae080)

**Comparison of two bundles for reducing surgical site infection in colorectal surgery: Multicentre cohort study**

**Authors:**

**Miriam Flores-Yelamos ^1,2, a^** <https://orcid.org/0000-0002-2640-9117>

[miriamfloresyelamos@gmail.com](mailto:miriamfloresyelamos@gmail.com)

**Aina Gomila-Grange ^3,a^** <https://orcid.org/0000-0001-6979-9269>

[agomila@tauli.cat](mailto:agomila@tauli.cat)

**Josep M Badia ^1,2^** <https://orcid.org/0000-0003-2928-5233>

[jmbadia@fphag.org](mailto:jmbadia@fphag.org)

**Alexander Almendral ^4^** <https://orcid.org/0000-0002-5459-3919>

[alexanderalmendral@iconcologia.net](mailto:alexanderalmendral@iconcologia.net)

**Ana Vázquez ^5^** <https://orcid.org/0000-0001-6732-1667>

[ana.vazquez@uab.cat](mailto:ana.vazquez@uab.cat)

**David Parés ^6^** https://orcid.org/0000-0001-8233-4888

[dapares@gmail.com](mailto:dapares@gmail.com)

**Marta Pascual ^7^** <https://orcid.org/0000-0003-0849-9048>

[mpascual@parcdesalutmar.cat](mailto:mpascual@parcdesalutmar.cat)

**Enric Limón ^4,8^** <https://orcid.org/0000-0002-5396-1521>

[elimon@iconcologia.net](mailto:elimon@iconcologia.net)

**Miquel Pujol ^9^** <https://orcid.org/0000-0002-6475-6208>

[mpujol@bellvitgehospital.cat](mailto:mpujol@bellvitgehospital.cat)

**Montserrat Juvany ^7^** <https://orcid.org/0000-0001-7385-278X>

mjuvanygomez@gmail.com

**and Members of the VINCat Colorectal Surveillance Team*, on behalf of VINCat Program****

**a:** **Authors with equal contribution**

1. Department of Surgery, Hospital General de Granollers, Granollers, Spain.
2. Universitat Internacional de Catalunya, Sant Cugat del Vallès, Barcelona, Spain
3. Department of Infectious Diseases, Hospital Universitari Parc Taulí, Sabadell, Spain.
4. Surveillance of Healthcare Related Infections in Catalonia Programme, VINCat, Catalonia, Spain.
5. Servei d'Estadística Aplicada, Universitat Autònoma de Barcelona, Bellaterra, Barcelona, Spain.
6. Colorectal Surgery Unit, Department of Surgery, Hospital Universitari Germans Trias i Pujol. Universitat Autónoma de Barcelona. Badalona, Barcelona, Spain.
7. Department of Surgery, Hospital del Mar, Barcelona, Spain.
8. Department of Public Health, Mental Health & Mother–Infant Nursing, Faculty of Nursing, University of Barcelona, Spain. CIBERINFEC, Instituto Carlos III, Madrid, Spain.
9. Department of Infectious Diseases, Hospital Universitari de Bellvitge - IDIBELL. L’Hospitalet de Llobregat, Spain. Centro de Investigación Biomédica en Red de Enfermedades Infecciosas (CIBERINFEC), Instituto de Salud Carlos III, Madrid, Spain. VINCat Program, Catalonia, Barcelona, Catalonia, Spain.

**Corresponding author.**

Josep M Badia

https://orcid.org/0000-0003-2928-5233

jmbadia@fphag.org

@BadiaJosepM

***Members the VINCat Colorectal Surveillance Team:**

**Domenico Fraccalvieri** https://orcid.org/0000-0003-3920-6199 Department of Surgery, Hospital Universitari de Bellvitge. L’Hospitalet de Llobregat, Spain. [dofrac@yahoo.es](mailto:dofrac@yahoo.es)

**Ana Abad-Torrent** https://orcid.org/0000-0002-5448-0113 Department of Anaesthesiology, Hospital Universitari Vall d’Hebrón, Barcelona, Spain. [ana.abad@vallhebron.cat](mailto:ana.abad@vallhebron.cat)

**Alejandro Solís-Peña** <https://orcid.org/0000-0001-5536-9559> Department of Surgery, Hospital Universitari Vall d’Hebrón, Barcelona, Spain. alejandro_solis85@hotmail.com

**Mireia Puig-Asensio** https://orcid.org/0000-0002-0722-2218 Department of Infectious Diseases, Hospital Universitari de Bellvitge. L’Hospitalet de Llobregat, Spain. Centro de Investigación Biomédica en Red de Enfermedades Infecciosas (CIBERINFEC, CB21/13/00009), Instituto de Salud Carlos III, Madrid, Spain. [mpuiga@bellvitgehospital.cat](mailto:mpuiga@bellvitgehospital.cat)

**Lucrecia López**, Infection control team. Hospital de Sant Joan Despí Moisès Broggi, Spain. [lucre.lopez@sanitatintegral.org](mailto:lucre.lopez@sanitatintegral.org)

**Marta Piriz**, <https://orcid.org/0000-0002-3166-4016> Infection control team. Hospital Universitari Sant Pau. Barcelona, Spain. [Mpirizm@santpau.cat](mailto:Mpirizm@santpau.cat)

**Mercè Hernández,** Department of Surgery, Hospital Universitari Parc Taulí, Sabadell, Spain. [mmhernandez@tauli.cat](mailto:mmhernandez@tauli.cat)

**** The members of this Program appear in Appendix 1.**

**Appendix 1. Members of Infection Control Teams participating in the program:**

Dolors Castellana and Elisa Montiu González, Hospital Universitari Arnau de Vilanova de Lleida; Graciano García Pardo and Francesc Feliu Villaró, Hospital Universitari Joan XXIII de Tarragona; Josep Rebull Fatsini and M. France Domènech Spaneda, Hospital Verge de la Cinta de Tortosa; Marta Conde Galí and Anna Oller Pérez-Hita, Hospital Universitari Dr. Josep Trueta Girona; Lydia Martín and Ana Lerida, Hospital de Viladecans; Sebastiano Biondo and Emilio Jiménez Martínez, Hospital Universitari de Bellvitge; Nieves Sopena Galindo and Ignasi Camps Ausàs, Hospital Universitari Germans Tries i Pujol; Carmen Ferrer and Luis Salas, Hospital Universitari Vall d’Hebron; Rafael Pérez Vidal and Dolors Mas Rubio, Althaia Xarxa Assistencial de Manresa; Irene García de la Red, Hospital HM Delfos; Mª Angels Iruela Castillo and Eva Palau i Gil, Clínica Girona; José Antonio Martínez Martínez and Mª Blanca Torralbo Navarro, Hospital Clínic de Barcelona; Maria López and Carol Porta, Hospital Universitari Mútua de Terrassa; Alex Smithson Amat and Guillen Vidal Escudero, Fundació Hospital de l'Esperit Sant; José Carlos de la Fuente Redondo and Montse Rovira Espés, Hospital Comarcal Mora d'Ebre; Arantxa Mera Fidalgo and Luis Escudero Almazán, Hospital de Palamós; Monserrat Ortega Raya and Aina Gomila, Hospital Parc Taulí de Sabadell; Vicens Diaz-Brito and Mª Carmen Álvarez Moya, Parc Sanitari Sant Joan de Déu (Hospital de Sant Boi); Laura Grau Palafox and Yésika Angulo Gómez, Hospital de Terrassa; Anna Besolí Codina and Carme Autet Ricard, Consorci Hospitalari de Vic; Carlota Hidalgo López and Marta Pascual Damieta, Hospital del Mar; Elisabeth Lerma-Chippirraz and Demelsa Maldonado López, Hospital General de Granollers; David Blancas and Esther Moreno Rubio, Consorci Sanitari del Garraf; Roser Ferrer i Aguilera, Hospital Sant Jaume de Calella; Simona Iftimie Iftimie and Antoni Castro-Salomó, Hospital Universitari Sant Joan de Reus; Rosa Laplace Enguídanos and Maria Carmen Sabidó Serra, Hospital de Sant Pau i Santa Tecla; Núria Bosch Ros, Hospital de Santa Caterina; Virginia Pomar Solchaga and Marta Piriz Marabaján, Hospital de la Santa Creu i Sant Pau; Laura Lázaro Garcia and Angeles Boleko Ribas, Hospital Universitari Quirón Dexeus; Jordi Palacín Luque and Alexandra Lucía Moise, Pius Hospital de Valls; Mª Carmen Fernández Palomares and Santiago Barba Sopeña, Hospital Universitari Sagrat Cor; Eduardo Sáez Huertas and Sara Burges Estada, Clínica Nova Aliança; Josep María Tricas Leris and Eva Redon Ruiz, Fundació privada Hospital de Mollet; Montse Brugués Brugués and Susana Otero Acedo, Consorci Sanitari de l'Anoia. Igualada; Maria Cuscó Esteve and Lourdes Gabarró, Hospital Comarcal de l'Alt Penedès; Fco. José Vargas-Machuca and Mª de Gracia García Ramírez, Centre MQ de Reus; Elena Vidal Díez and Ana Maria Ciscar Bellés, Consorci Hospitalari del Maresme. Hospital de Mataró; Mariló Marimón Morón and Marisol Martínez Sáez, Hospital Universitari General de Catalunya; Josep Farguell and Mireia Saballs, QUIRON Salud; Montserrat Vaqué Franco and Leonor Invernón Garcia, Hospital de Barcelona; Rosa Laplace Enguídanos and Meritxell Guillemat Marrugat, Hospital Comarcal del Vendrell; Ana Coloma Conde and Lucrecia López González, Hospital Moisès Broggi.

**Supplementary Materials - Index**

| **Supplementary Figures and Tables** |  |
| --- | --- |
| **Supplementary Table 1:** Inclusion and exclusion criteria for colorectal surgery surveillance. | *page 5* |
| **Supplementary Table 2:** Prophylactic measures for SSI included in each bundle.  MIS: minimally invasive surgery. | *page 6* |
| **Supplementary Table 3.** Adherence rate to the preventive measures included in the groups of study.  ND: no data. | *page 7* |
| **Supplementary Table 4:** Effect of the individual preventive measures contained in the bundles on incisional SSI rates (adding superficial and deep categories).  OR: odds ratio, 95% CI: 95% confidence interval. | *page 8* |
| **Supplementary Figure 1:** Time line showing the three periods analysed and the bundle interventions carried out in each one.  ATB: antibiotic; OAP: oral antibiotic prophylaxis, MBP: mechanical bowel preparation; CHG-alcohol: chlorhexidine gluconate in alcohol. | *page 9* |
| **Supplementary Figure 2:** Chart showing the balance of covariates. The balance is assessed by the value of standardized mean differences (SMD), which represents the difference of the mean of each covariate between the standardized treatment groups. SMD values ​​close to zero or below the limit indicate a good balance, while higher values ​​indicate a poor balance.  IPTW: inverse probability of treatment weighting | *page 9* |

**Supplementary Figures and Tables**

**Supplementary Table 1:** Inclusion and exclusion criteria for colorectal surgery surveillance.

| **Inclusion criteria** |
| --- |
| Colorectal elective resection surgery (malignant and benign diseases were included). |
| Clean-contaminated (wound class 2) and contaminated (wound class 3) cases. |
| Patients with multiple procedures during the same surgical intervention from 2016 onwards. |
| **Exclusion criteria** |
| Emergency surgery |
| Presence of peritonitis (wound class 4). |
| Patients with multiple procedures during the same surgical intervention until 2015. |
| Patients with previous ostomies. |
| Hospitals that performed fewer than 10 procedures annually. |
| Patients aged under 18. |

**Supplementary Table 2:** Prophylactic measures for SSI included in each bundle.

| **Bundle 1** | |
| --- | --- |
| Adequate systemic iv antibiotic prophylaxis | All following items must be fulfilled: |
|  | - Start 30-60min before incision. |
|  | - Intraoperative re-dosing when indicated. |
|  | - Do not prolong >24h |
|  | Recommended: |
|  | - Metronidazole 15mg/kg + gentamycin 5mg/kg |
|  | - Cefuroxime 1,5g + metronidazole 15mg/kg |
|  | - Cefazolin 2g + metronidazole 15mg/kg |
|  | - Amoxicillin-clavulanate 2g |
| Minimally invasive surgery | When indicated (laparoscopic or robotic) |
| Oral antibiotic prophylaxis | Recommended: |
|  | - Metronidazole 750mg + neomycin 1g (three doses the day before the procedure) |
|  | - Erythromycin 1g + metronidazole 750 mg (three doses the day before the procedure) |
| Mechanical bowel preparatiol | The day before the surgery |
| Use of double-ring plastic wound edge retractor | In open or minimally invasive surgery |
| Maintenance of normothermia | Goal > 36ºC at the end of surgery |
| **Bundle 2** | |
| Six measures of Bundle-1 plus all the following measures: | |
| Adequate hair management | No skin hair removal or removal with single-use electric clippers |
| Skin antisepsis with CHG-alcohol | Decontamination of intact skin with 2% chlorhexidine gluconate in 70% isopropyl alcohol |
| Change of instruments before wound closure | Exchange of gloves, surgical sices and material (as vaccum cleaner, lamp handles, electric scalpel, etc) |
| Maintenance of normoglycemia | Target blood glucose levels around 150-200mg/dL in diabetic and non-diabetic patients |

**Supplementary Table 3.** Adherence rate to the preventive measures included in the groups of study.

ND: no data

|  | **Measures** | **Baseline group** | **Bundle 1** | **Bundle 2** |
| --- | --- | --- | --- | --- |
| **Colon Surgery** | | | | |
| Baseline group measures | Adequate antibiotic prophylaxis | 11,476 (87.5%) | 2,322 (81.9%) | 6,254 (85.3%) |
|  | Minimally invasive surgery | 7,723 (58.9%) | 2,141 (75.5%) | 5,827 (79.5%) |
| Bundle 1 specific measures | Oral antibiotic prophylaxis | ND | 1,664 (79.7%) | 6,836 (98.4%) |
|  | Mechanical bowel preparation | ND | 1,931 (72.6%) | 4,980 (79.6%) |
|  | Double-ring wound retractor | ND | 2,043 (75.9%) | 5,132 (76.8%) |
|  | Maintenance of normothermia | ND | 1,995 (77.0%) | 5,582 (88.6%) |
| Bundle 2 specific measures | Adequate hair removal | ND | ND | 2,981 (97.8%) |
|  | 2% chlorhexidine in alcohol | ND | ND | 6,182 (88.8%) |
|  | Glycaemic control | ND | ND | 4,690 (64.0%) |
|  | Changing of surgical instruments | ND | ND | 428 (91.8%) |
| **Rectal Surgery** | | | | |
| Baseline group measures | Adequate antibiotic prophylaxis | 4,790 (86.3%) | 865 (80.5%) | 1,964 (85.2%) |
|  | Minimally invasive surgery | 3,263 (58.8%) | 800 (74.5%) | 1,933 (83.9%) |
| Bundle 1 specific measures | Oral antibiotic prophylaxis | ND | 590 (80.4%) | 2,153 (98.3%) |
|  | Mechanical bowel preparation | ND | 819 (81.0%) | 1,780 (89.1%) |
|  | Double-ring wound retractor | ND | 976 (95.2%) | 1,917 (91.4%) |
|  | Maintenance of normothermia | ND | 569 (62.9%) | 1,430 (75.3%) |
| Bundle 2 specific measures | Adequate hair removal | ND | ND | 1,005 (98.0%) |
|  | 2% chlorhexidine in alcohol | ND | ND | 1,750 (80.3%) |
|  | Glycaemic control | ND | ND | 1,391 (60.4%) |
|  | Changing of surgical instruments | ND | ND | 101 (86.3%) |

**Supplementary Table 4:** Effect of the individual preventive measures contained in the bundles on incisional SSI rates (adding superficial and deep categories).

OR: odds ratio, 95% CI: 95% confidence interval.

|  | | | | |
| --- | --- | --- | --- | --- |
|  | **Univariate** | | **Multivariate** | |
| **Bundle measures** | **OR [95% CI]** | **P** | **OR [95% CI]** | **P** |
| **Colorectal surgery** | | | | |
| Adequate antibiotic prophylaxis | 0.92 [0.82 to 1.04] | 0.163 | 0.93 [0.83 to 1.05] | 0.217 |
| Minimally invasive surgery | 0.44 [0.40 to 0.48] | <.001 | 0.53 [0.48 to 0.58] | <.001 |
| Oral antibiotic prophylaxis | 0.34 [0.30 to 0.39] | <.001 | 0.57 [0.46 to 0.70] | <.001 |
| Mechanical bowel preparation | 0.39 [0.35 to 0.44] | <.001 | 1.08 [0.88 to 1.33] | 0.462 |
| Double-ring wound retractor | 0.33 [0.29 to 0.38] | <.001 | 0.60 [0.50 to 0.71] | <.001 |
| Maintenance of normothermia | 0.39 [0.35 to 0.44] | <.001 | 0.93 [0.76 to 1.14] | 0.491 |
| Adequate hair removal | 0.47 [0.39 to 0.56] | <.001 | 1.13 [0.91 to 1.39] | 0.266 |
| 2% chlorhexidine in alcohol | 0.37 [0.33 to 0.42] | <.001 | 0.73 [0.59 to 0.90] | 0.003 |
| Glycemic control | 0.41 [0.36 to 0.48] | <.001 | 0.96 [0.78 to 1.18] | 0.682 |
| Changing of surgical instruments | 0.58 [0.37 to 0.87] | 0.014 | 1.52 [0.94 to 2.32] | 0.069 |
| **Colon surgery** | | | | |
| Adequate antibiotic prophylaxis | 0.91 [0.79 to 1.05] | 0.174 | 0.91 [0.79 to 1.05] | 0.192 |
| Minimally invasive surgery | 0.42 [0.38 to 0.46] | <.001 | 0.51 [0.45 to 0.56] | <.001 |
| Oral antibiotic prophylaxis | 0.27 [0.23 to 0.32] | <.001 | 0.44 [0.34 to 0.56] | <.001 |
| Mechanical bowel preparation | 0.34 [0.30 to 0.40] | <.001 | 1.05 [0.83 to 1.34] | 0.667 |
| Double-ring wound retractor | 0.32 [0.28 to 0.37] | <.001 | 0.65 [0.52 to 0.80] | <.001 |
| Maintenance of normothermia | 0.37 [0.32 to 0.43] | <.001 | 0.96 [0.75 to 1.23] | 0.771 |
| Adequate hair removal | 0.44 [0.36 to 0.55] | <.001 | 1.06 [0.81 to 1.37] | 0.668 |
| 2% chlorhexidine in alcohol | 0.36 [0.31 to 0.42] | <.001 | 0.75 [0.58 to 0.97] | 0.026 |
| Glycaemic control | 0.41 [0.34 to 0.48] | <.001 | 1.02 [0.79 to 1.32] | 0.871 |
| Changing of surgical instruments | 0.50 [0.28 to 0.82] | 0.011 | 1.27 [0.70 to 2.15] | 0.395 |
| **Rectal surgery** | | | | |
| Adequate antibiotic prophylaxis | 0.96 [0.79 to 1.18] | 0.719 | 0.97 [0.80 to 1.19] | 0.771 |
| Minimally invasive surgery | 0.49 [0.42 to 0.57] | <.001 | 0.56 [0.48 to 0.66] | <.001 |
| Oral antibiotic prophylaxis | 0.49 [0.40 to 0.59] | <.001 | 0.97 [0.65 to 1.48] | 0.888 |
| Mechanical bowel preparation | 0.48 [0.40 to 0.57] | <.001 | 0.89 [0.58 to 1.34] | 0.577 |
| Double-ring wound retractor | 0.40 [0.31 to 0.50] | <.001 | 0.61 [0.45 to 0.83] | 0.002 |
| Maintenance of normothermia | 0.46 [0.37 to 0.55] | <.001 | 0.84 [0.59 to 1.18] | 0.310 |
| Adequate hair removal | 0.54 [0.40 to 0.72] | <.001 | 1.20 [0.82 to 1.71] | 0.338 |
| 2% chlorhexidine in alcohol | 0.43 [0.33 to 0.55] | <.001 | 0.78 [0.55 to 1.11] | 0.166 |
| Glycaemic control | 0.46 [0.34 to 0.59] | <.001 | 0.85 [0.59 to 1.21] | 0.366 |
| Changing of surgical instruments | 0.94 [0.42 to 1.82] | 0.867 | 2.14 [0.92 to 4.33] | 0.051 |

**Supplementary Figure 1:** Time line showing the three periods analysed and the bundle interventions carried out in each one.

**
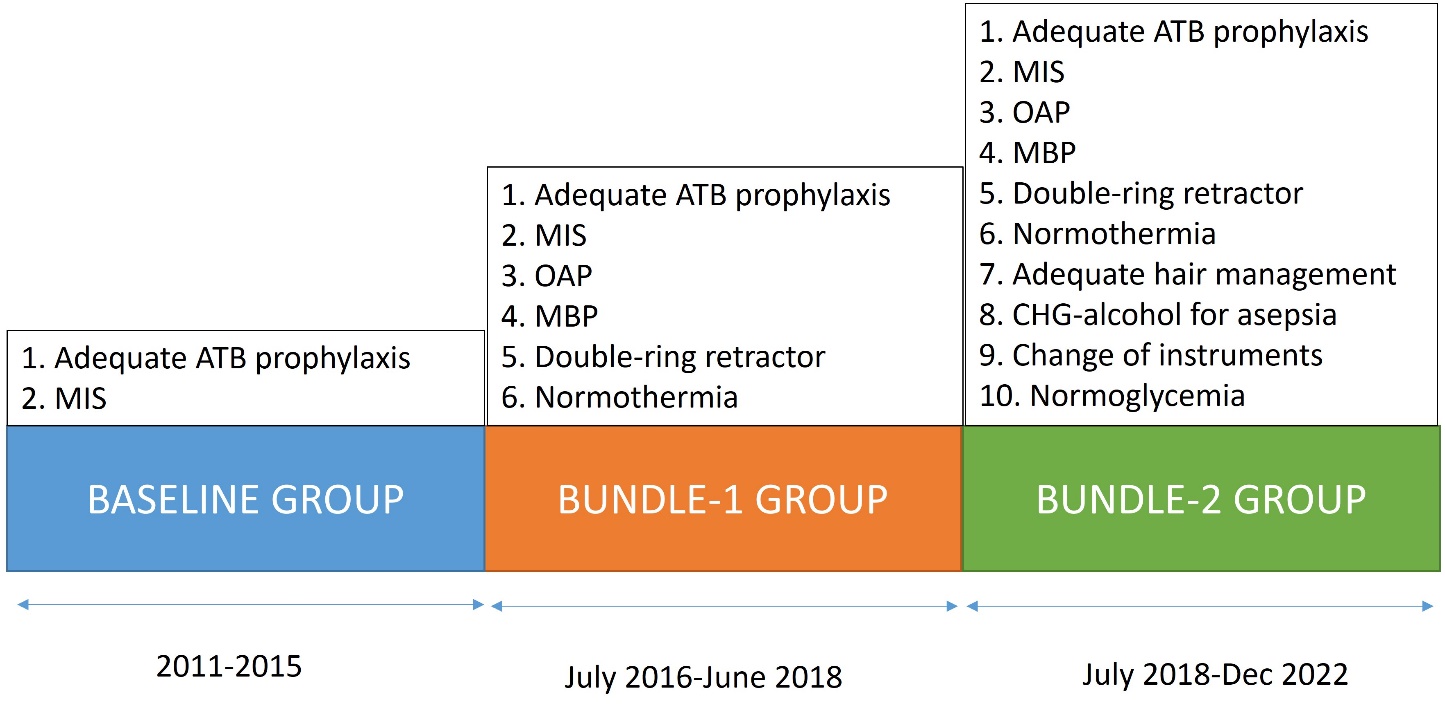
**

**Supplementary Figure 2:** Chart showing the balance of covariates. The balance is assessed by the value of standardized mean differences (SMD), which represents the difference of the mean of each covariate between the standardized treatment groups. SMD values ​​close to zero or below the limit indicate a good balance, while higher values ​​indicate a poor balance.

IPTW: inverse probability of treatment weighting


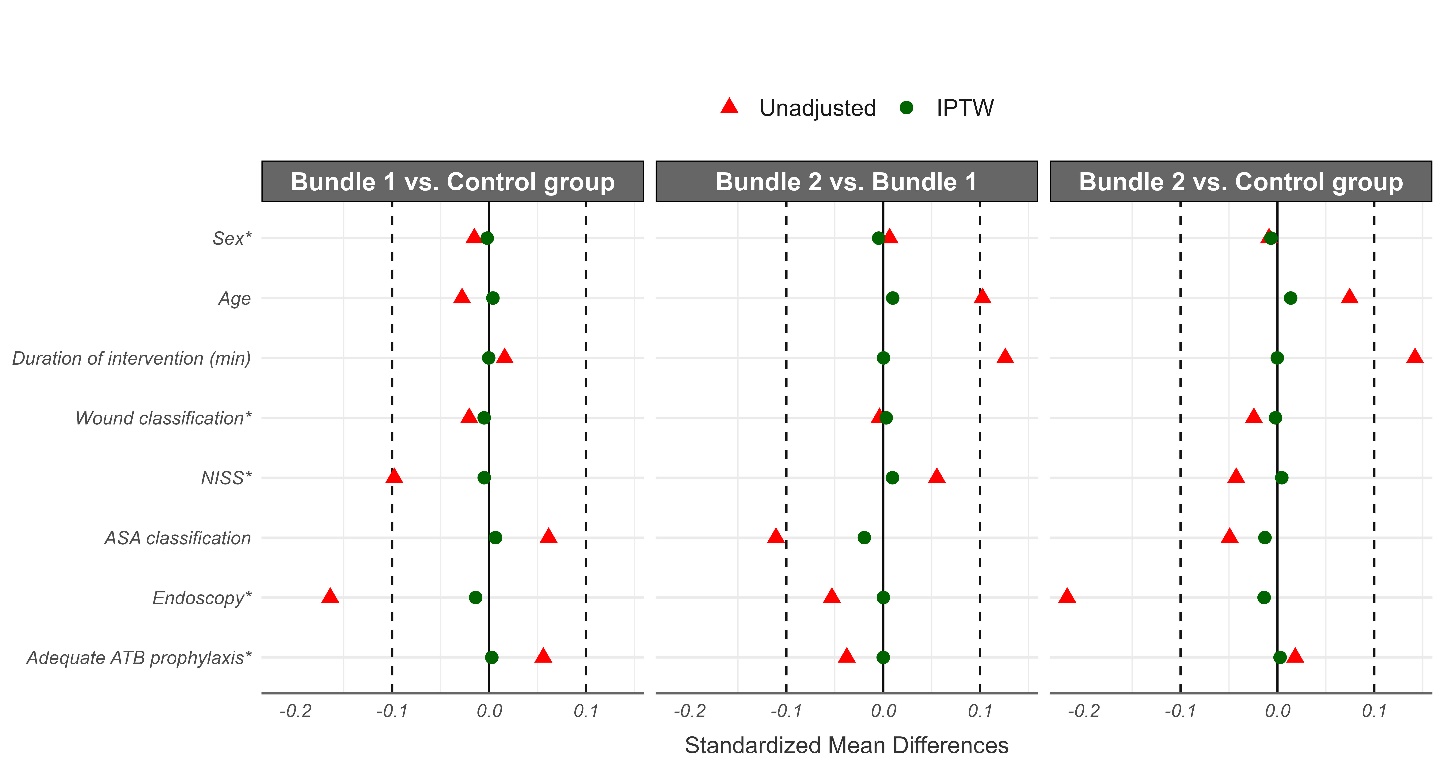

Supplement: zrae080_Supplementary_Data [file zrae080_supplementary_data.docx]
